# PReS-FINAL-2351: Children with probable SLE by ACR criteria may need more aggressive lupus treatment early in the disease course

**DOI:** 10.1186/1546-0096-11-S2-P341

**Published:** 2013-12-05

**Authors:** A Patwardhan, I Dvorchik, C Spencer, Probable Lupus

**Affiliations:** 1 Pediatric Rheumatology, University of Misouri School of Medicine, Columbia, OH, USA; 2 Pediatric Rheumatology, Ohio State University, Columbus, OH, USA; 3 Biostatistics, Nationwide Childrens Hospital, Ohio State University, Columbus, OH, USA; 4 Pediatric Rheumatology, Nationwide Childrens Hospital, Ohio State University, Columbus, OH, USA

## Introduction

The pediatric lupus patients are required to meet the American College of Rheumatology (ACR) minimum criteria to be included in the research cohort and considered for aggressive therapy. This approach may delay early aggressive therapy.

## Objectives

The research explores whether by delaying diagnosis until 4/11 criteria are met affects the patient outcome negatively. To our knowledge there is no published literature of the "probable" pediatric systemic lupus erythematosus (pSLE) population.

## Method

Institutional Review Board approval was obtained to retrospectively review the charts of 98 SLE patients seen in the pediatric rheumatology clinic at Nationwide Children's Hospital over the past 24 years. All the patients were divided in to two groups, 'definitive pSLE' - who met the minimum 4/11, or more ACR criteria and the 'probable pSLE' who did not meet the minimum criteria at presentation in rheumatology clinic. Both the groups were assessed for disease severity, damage and gradient of damage. Appropriate statistical tests were used i.e. Chi-Square test, Fisher's Exact test, Univariate logistic regression and Wilcoxon two-sample test were used for statistics for various data set. All tests were conducted in SAS 9.2

## Results

Out of 98 pSLE patients 71% were included in definitive pSLE (D pSLE) group while 28.57% were included in probable pSLE (P pSLE) group.The mean time for PpSLE group to reach D pSLE status was 20.3 months. There was no difference in the ethnic distribution (p = 0.7370) in D pSLE and PpSLE. PpSLE were more likely to have higher male: female ratio (p = 0.032), and were older at presentation that D pSLE (p = 0.045).PpSLE patients were less likely to have internal organ involvement (7.1% Vs 25.7%), were less likely to be hospitalized and receive pulse steroids (p = 0.0142) or oral steroids (0.0172) at presentation. PpSLE patients were less likely to be hospitalized receive pulse steroids ever (p = 0.0628), were less likely to have renal disease ever (p = 0.0653) and nervous system disease ever (p = 0.0182). Probable SLE was more likely to receive hydroxychloroquine (p = 0.050).The organ damage was assessed using SLICC/ACR damage index at 1, 5 and 10 years post diagnosis. The maximum damage was recorded within first 5 years of the diagnosis. Initial damage was predictive of later damage. D pSLE had higher disease damage scores at 5 and 10 years We compared the gradient between the onsets of symptoms and the development of organ damage in the two groups. The PpSLE patients had significantly higher internal organ damage gradient as compared to D pSLE (p value = 0.0169)

## Conclusion

In our population PpSLE patients had a significantly high gradient of damage than the D pSLE group.In spite of D pSLE being more severe diseases ever and more diseases damage, the disease damage progression was steeper and faster in PpSLE. This can only be explained by the fact that PpSLE patients received less intense treatment at presentation than D pSLE group. It may be that PpSLE patients at presentation need just as vigorous treatment as the children with definitive SLE

## Disclosure of interest

None declared.

